# Knock-down of Kaiso induces proliferation and blocks granulocytic differentiation in blast crisis of chronic myeloid leukemia

**DOI:** 10.1186/1475-2867-12-28

**Published:** 2012-06-18

**Authors:** Jaime Cofre, João R L Menezes, Luciana Pizzatti, Eliana Abdelhay

**Affiliations:** 1 Laboratório de Embriologia Molecular e Câncer, Universidade Federal de Santa Catarina, Sala 313b, CEP 88040-900, Florianópolis, SC, Brazil; 2 Laboratório de Neuroanatomia Celular, Programa de Anatomia, Instituto de Ciências Biomédicas, Universidade Federal do Rio de Janeiro, Rio de Janeiro, Brazil; 3 Divisão de Laboratórios do CEMO, Instituto Nacional do Câncer, Rio de Janeiro, Brazil

## Abstract

**Background:**

Kaiso protein has been identified as a new member of the POZ-ZF subfamily of transcription factors that are involved in development and cancer. There is consistent evidence of the role of Kaiso and its involvement in human tumorigenesis but there is no evidence about its role in hematopoietic differentiation or establishment of chronic myeloid leukemia (CML). We used, normal K562 cell line, established from a CML patient in blast crisis, and imatinib-resistant K562 cell line, to investigate the specific distribution of Kaiso and their contribution to the cell differentiation status of the blast crisis of CML (CML-BP).

**Results:**

We found cytoplasmic expression of Kaiso, in K562 cells and patients, confirmed by immunofluorescence, immunohistochemistry and western blot of cytoplasmic protein fraction. Kaiso was weakly expressed in the imatinib-resistant K562 cell line confirmed by immunofluorescence and western blot. The cytoplasmic expression of Kaiso was not modified when the K562 cells were treated for 16 h with imatinib 0.1 and 1 μM. In our study, small interfering RNA (siRNA) was introduced to down regulate the expression of Kaiso and p120ctn in K562 cell line. Kaiso and p120ctn were down regulated individually (siRNA-Kaiso or siRNA-p120ctn) or in combination using a simultaneous co-transfection (siRNA-Kaiso/p120ctn). We next investigated whether knockdown either Kaiso or p120ctn alone or in combination affects the cell differentiation status in K562 cells. After down regulation we analyzed the expression of hematopoietic cell differentiation and proliferation genes: SCF, PU-1, c-MyB, C/EBPα, Gata-2 and maturation markers of hematopoietic cells expressed in the plasma membrane: CD15, CD11b, CD33, CD117. The levels of SCF and c-MyB were increased by 1000% and 65% respectively and PU-1, Gata-2 and C/EBPα were decreased by 66%, 50% and 80% respectively, when Kaiso levels were down regulated by siRNA. The results were similar when both Kaiso and p120ctn were down regulated by siRNA. The increased expression of SCF and decreased expression of GATA-2 could be responsible by the higher cell viability detected in K562 cells double knock-down of both Kaiso and p120ctn. Finally, we studied the effect of knock-down either Kaiso or p120ctn, alone or in combination on CD15, CD11b, CD33 and Cd117 expression. Using siRNA approach a reduction of 35%, 8% and 13% in CD15, CD33 and CD117 levels respectively, were achieved in all transfections, when compared to scrambled knock-down cells.

**Conclusion:**

These results suggest that both Kaiso and p120ctn, contributes to maintaining the differentiated state of the K562 cells and similar to other cancers, cytoplasmic localization of Kaiso is related to a poor prognosis in CML-BP. By the broad and profound effects on the expression of genes and markers of hematopoietic differentiation produced by Kaiso knock-down, these findings reveal Kaiso as a potential target for selective therapy of CML.

## Background

Chronic myeloid leukemia (CML) is a clonal disorder of the pluripotent hematopoietic stem cell (resulting in a progressive granulocytosis), in which a reciprocal translocation t(9;22)(q34;q11) forms a Philadelphia (Ph) chromosome and creates a novel fusion gene, bcrabl [1]. Its corresponding protein has a constitutively activated tyrosine kinase that is central to the pathogenesis of CML [2].

The disease follows a triphasic course: an initial chronic phase (CP) lasting 3–5 years, an accelerated phase (AP) lasting 6–18 months and the final phase called blast crisis (BC) or acute leukemia, defined hematologically by the increase of leukemic blasts (myeloid or lymphoid) in peripheral blood and/or bone marrow (more than 20%). At this stage of the disease, many patients died between three and six months, because they are refractory to most treatments, including resistance to imatinib [3].

Imatinib has emerged as the leading compound to treat CML. It targets the ATP-binding site of different tyrosine kinases including bcr-abl, the platelet-derived growth factor receptor [4], and C-KIT [5]. Imatinib selectively induces growth arrest and apoptosis of bcr-abl-positive leukemia cells with minimal effect on normal hematopoietic progenitors [6-8]. Of note, this agent has proven very effective in patients in chronic phase of CML [9] and to a lesser extent, in patients in accelerated phase and blast crisis [7]. Although treatment with imatinib achieves complete hematologic remission in the great majority of patients with CML, total cytogenetic and molecular responses are relatively rare events [10].

It has become widely accepted that activation of the bcr-abl tyrosine kinase is causative for CML [11]. Still, involvement of additional molecular events in the pathogenesis of CML has been demonstrated [12]. For instance, in BC of CML elevated levels of β-catenin lead to expansion of the granulocyte-macrophage progenitor (GMP) subset [12], and inactivation of the transcription factor JunB is able to increase the number of long-term hematopoietic stem cells (LT-HSC) and GMP in a murine model of myeloproliferative disease [13].

Several recent studies about the participation of Kaiso in the β-catenin regulation have been obtained, when it has been found that Kaiso inhibits activation mediated by β-catenin of the Mmp7 gene (also known as matrilysin), which is well known for metastatic spread [14]. Another study suggests that Kaiso can regulate TCF/LEF1-activity, via modulating HDAC1 and β-catenin-complex formation [15]. This shows that Kaiso can directly regulate the signaling pathway of canonical Wnt/β-catenin widely known for its involvement in human tumors. Other evidence also showed that Kaiso rescues the dorsalization of the mesoderm produced by β-catenin and siamois in Xenopus laevis [16]. Siamois is a high mobility group (HMG)-box transcription factor that promotes the dorsalization of the mesoderm of amphibians and is a well-known target of the canonical Wnt pathway involving TCF/LEF. The Kaiso overexpression decreases the ability of TCF/LEF to interact with β-catenin, which implies that Kaiso and TCF/LEF are associated in the nucleus [17]. Despite this evidence the role of Kaiso in hematopoiesis has not been explored.

Who is Kaiso? Kaiso protein (encoded by the zinc finger and broad-complex, tramtrack and bric-a-brac (BTB)-domain-containing 33 gene ZBTB33) is a transcriptional factor that has a BTB/POX domain for the protein-protein interaction in the amino-terminal portion and a “Zinc Finger” domain for interaction with DNA in the carboxyl-terminal portion [18,19]. Due to the aforementioned characteristics Kaiso is member of a subfamily of “zinc finger” proteins known as POZ-ZF [19].

Most members of this subfamily (POZ-ZF) transcriptional factors including, Kaiso, BCL6, PLZF, HIC-1, FAZF, APM1, MIZ-1, ZBTB7 and champignon are involved in the process of cancer development [20-26].

Kaiso protein interacts specifically with p120 catenin (p120ctn), a member of the armadillo family that owns β-catenin [19]. β-catenin and p120ctn are very similar molecules possessing the two i. domains of interaction with the cytosolic portion of cadherins and ii. the ability to translocate from the cytoplasm to the nucleus [27]. A p120ctn is a regulator of the kaiso function and it is known that in the nucleus of the cell they directly modulate the action of canonical Wnt pathways and target genes of β-catenin, which is another indication of the importance of Kaiso in the development of cancer [28].

The genes transcriptionally regulated by Kaiso are matrilysin [14], c-myc and cyclin D1 [17], all of them widely known for their involvement in cell proliferation and metastasis and all also regulated by the domain “Zinc finger” of Kaiso [28]. Gene Wnt11 is another important and well- known regulatory target, which belongs to the non-canonical Wnt pathways [29].

The Kaiso protein, unlike other members of the subfamily, appears to be the only factor with bimodal features in their interaction with DNA, being able to interact specifically with methylated CpG island sites and with consensus DNA sequences CTGCNA [30,31]. Kaiso apparently recognize methylated DNA by a canonical mechanism [32] and their epigenetic function has been widely described as a transcriptional repressor (revised in [33]). This recognition of DNA methylation is important for the epigenetic silencing of tumor suppressor genes, which is an essential role of Kaiso in colon cancer development processes [34]. A breakthrough in understanding how methylation-mediated repression worked was the finding that Kaiso interacts with a co-repressor complex containing histone deacetylase (HDAC). Regarding epigenetic silencing, the Kaiso protein also acts as a histone-deacetylase-dependent transcriptional repressor [28]. The HDAC catalyzes the deacetylation of histones and these changes facilitate more closed chromatin conformation and restrict gene transcription. The HDAC acts as a protein complex with corepressors recruited. Some of them are directly recruited by Kaiso as NCOR1 (nuclear receptor co-repressor 1) [35] and SIN3A [17].

Recently a clinic study has shown for the first time that the subcellular localization of Kaiso in the cytoplasm of a cell is directly associated with the poor prognosis of patients with lung cancer [36]. Such data shows a direct relationship between the clinical profile of patients with pathological expression of Kaiso. Therefore, evidence of changes in subcellular localization seems to be relevant to the diagnosis and prognosis of lung tumors.

Despite the growing number of experimental data demonstrating the direct regulatory role of Kaiso on: (i) canonical Wnt pathways, activation of β-catenin and deregulation of the Wnt signaling pathways, it is considered today as a common phenomenon in cancer and leukemia [37], (ii) non-canonical Wnt pathways, Wnt11 is directly regulated by β-catenin and Kaiso [15], (iii) the role of Kaiso in tumorigenesis and (iv) the direct relationship between cytoplasmic Kaiso and the clinical profile of disease [36], there are no data on the involvement of Kaiso in hematopoiesis and CML and also there are no data linking Kaiso with the blast crisis of the disease.

We studied the localization and the role of Kaiso in the cell differentiation status of the K562 cell line, established from a CML patient in blast crisis. Using western blot and immunofluorescence we found for the first time, the cytoplasmic distribution of kaiso in CML-BP cells, and consistent with the poor prognosis on the acute phase of the disease. The imatinib-resistant K562 cells showed a significant reduction in the cytoplasmic Kaiso expression. We next investigated, through siRNA, whether knock-down either Kaiso or p120ctn alone or in combination affects the cell differentiation status of K562 cells. We quantified the levels of hematopoietic cell differentiation and proliferation genes: SCF, c/EBPα, c-Myb, GATA-2, PU.1, Wnt11, by QRT-PCR and (ii) maturation markers of hematopoietic cells such as CD15, CD11b, CD33 and CD117, by FACS analysis. We found that knock-down of either Kaiso or p120ctn alone or combination decreased PU-1, C/EBPα, Gata-2 and increased SCF and c-MyB levels. Also, the combined Kaiso and P120ctn knock-down had a 51% induction in cell proliferation compared to the scrambled knock-down cells. The Kaiso or P120ctn knock-down alone or double knock-down decreased CD15, CD33 and CD117 levels when compared to scrambled knock-down cells. Taken together, these results suggest that Kaiso and p120ctn contributes to maintaining the undifferentiated state of the CML-BP and Kaiso seems to be a central molecule involved in broad regulation of differentiation and proliferation genes in CML-BP and also probably related to imatinib resistance.

## Materials and methods

### Cell line

K562 and LAMA-84 cell line were maintained in RPMI 1640 medium supplemented with 10% foetal bovine serum (Hyclone), 100 U/ml penicillin (Invitrogen), 100 mg/mL streptomycin (Invitrogen) at 37°C in 5% CO2. K562, established from a CML patient in blast crisis [38], was used as a BCR-ABL-positive cell line. Imatinib-resistant K562 cell line (a gift from A. Mencalha, INCA RJ) was obtained by in vitro passaging of K562 in progressively increasing doses of imatinib. LAMA-84 is a human leucocytic cell line with basophilic characteristic [39].

### Bone marrow samples

All samples were obtained from patients admitted to or registered at the Instituto Nacional de Câncer (Rio de Janeiro, Brazil), following the guidelines of the local Ethics Committee and the Helsinki declaration. Diagnoses and follow-up were based on hematologic, cytogenetic and molecular assays.

### Drug treatment

K562 cell line were exposed to different doses of Imatinib dissolved in Dimethyl sulphoxide (DMSO; Sigma Aldrich). DMSO-treated cells were used as vehicle controls.

### Viability determination

The viability of cells was measured using a 4-[3-(4-Iodophenyl)-2-(4-nitrophenyl)-2 H-5-tetrazolio]-1,3-benzene disulphonate (WST-1) assay (Roche). Approximately 2 × 10^5^cells/mL. Cells were plated into 96-well microplates (Corning) for 24 h. After 24 h, 10 μL WST-1 was added to each well, and plates were incubated at 37°C for an additional 2 h. Plates were read on a microplate reader (Bio-Rad, model 550) at 450 nm with a reference wavelength at 630 nm.

### RNAi knockdown and transfection

All RNA oligonucleotides described in this study were synthesized and purified using highperformance liquid chromatography (HPLC) at Integrated DNA Technologies (Coralville, Iowa), and the duplex sequences are available upon request. RNAi knockdown and transfections were performed following the manufacturer’s protocols of the TriFECTa Dicer-Substrate RNAi kit (Integrated DNA Technologies, Coralville, IA) and the CodeBreaker siRNA Transfection Reagent (Promega,USA). K562 cells (1 × 10^6^ cells per well) were split in 24-well plates to 60% confluency in RPMI media 1 day prior to transfection. The TriFECTa kit contains control sequences for RNAi experiments which include a fluorescent-labeled transfection control duplex and a scrambled universal negative control RNA duplex that is absent in human, mouse, and rat genomes. Fluorescence microscopy and FACS monitored the transfection efficiency according to the manufacturer’s recommendations. Only experiments in which transfection efficiencies were ≥ 90% were evaluated. RNA levels were measured 36 h after transfection, and protein levels were measured 80 h later. All duplexes used were evaluated at 25, 10, 1, and 0.1 nM. All transfections were minimally performed in triplicate, and the data were averaged. Knockdown of Kaiso and P120ctn was performed, and RNA, protein extraction, QRT-PCR, Western blot, and FACS analysis were done as described above.

### Real time PCR

QRT-PCR Analysis Quantitation of Kaiso, P120ctn, Wnt11, β-catenin, SCF, c-MYB, c-EBPα, Gata-2, PU-1 RNA transcripts was carried out by real time PCR (QRT-PCR). Two micrograms of total RNA from K562 cell line or transfected K562 cell line, were reverse transcribed with Superscript III Reverse transcriptaseVR (Invitrogen). cDNAs were mixed with SYBR Green PCR Master MixVR (Applied Biosystems) and specific primers. Real time PCR was performed in an ABI Prism 7000 thermocycler (Applied Biosystems), with 50 cycles of 15 s at 95°C and 2 m at 68°C. Expression levels were estimated in triplicate with specific and control primers. For each sample, the relative amounts of transcripts of the target gene and the internal control were estimated from a standard curve. Results were expressed in arbitrary units as the ratio of the target gene transcript/internal transcript (data represented by average ± SD of three measurements).

### Western blot analysis

Protein lysates were prepared as previously reported [40]. Protein concentrations were determined by the Bradford method. Approximately 200 μg protein was resolved on 7% sodium dodecyl sulfate-polyacrylamide gel electrophoresis (SDS-PAGE) gels, blotted onto nitrocellulose membranes (Bio-Rad, CA) and probed with individual antibodies, and visualized by the enhanced chemiluminescence ECL Plus Western Blotting Detection ReagentsVR (GE healthy care, United Kingdom). The following antibodies were used: anti-kaiso (Santa Cruz Biotechnology, Santa Cruz, CA), anti-actin (Santa Cruz Biotechnology, Santa Cruz, CA). The secondary antibodies were horseradish peroxidase (HRP)-conjugated rabbit antimouse IgG (Santa Cruz Biotechnology, Santa Cruz, CA).

### Immunofluorescence and FACS analysis

K562 cells were incubated in RPMI (Invitrogen, USA), harvested after 16 h, and washed several times in PBS. Normal and imatinib-resistant K562 cells were resuspended at a concentration of 2 × 10^6^/ml in PBS. Normal and imatinib-resistant K562 cells (50,000) were attached to microscope slides by centrifugation for 2 min at 800 rpm at high acceleration in a Cytospin 2 centrifuge (Shandon; Frankfurt, Germany) and dried for 10 min at 37°C in a sterilizer. For immunofluorescence, culture cell were prefixed in formaldehyde vapor by placing the slide into a chamber containing paper towel embedded with formaldehyde for 10 min. Subsequently, the slides were immersed in buffered 4% paraformaldehyde for 15 min. After several washes in phosphate-buffered saline (PBS; pH 7.4), K562 cells were incubated for 72 h at 4°C with primary antibodies diluted in PBS with 0.3% Triton-X 100 (Reagen, Curitiba, PR, Brazil) and 5% normal goat serum (Invitrogen, Carlsbad, CA). Primary antibodies were the following: anti-Kaiso (mouse 1:100; Santa Cruz, CA), anti β-tubulin (mouse 1:500; Sigma), Secondary antibodies were incubated for 2 h at room temperature. Secondary antibodies were the following: goat anti-mouse IgG conjugated with Cy3 (1:800; Jackson ImmunoResearch). Slides were counterstained with DAPI (Molecular Probes). Conventional fluorescence microscopy was performed in an Eclipse TE200 inverted microscope (Nikon, Tokyo, Japan), equipped with a CoolSNAP-Pro cf CCD camera(Media Cybernetics, Silver Spring, MD; monochrome). Images were acquired with the aid of Image-Pro Express software (version 4.5.1.3) and edited with Photoshop CS5.1 (Adobe, San Jose, CA). For FACS analysis, antibodies that recognize cell surface myeloid-specific antigens GPA-phycoerythrin (PE), CD33-fluorescein isothiocyanate (FITC), CD11b-PE, CD15-FITC, CD117-PE, CD71-FITC (Becton Dickinson) were used. Appropriated isotype-matched controls (Becton Dickinson) were used.

### Immunohistochemistry

Immunohistochemical staining was performed in formalin-fixed, paraffin-embedded bone marrow slides from five CML patients in the chronic phase and six patients in the blastic phase, according to standard procedures. Heat-induced epitopes were retrieved in Tris buffer (pH = 9.9; Dako, Denmark) in a microwave processor. Tissue sections were subsequently incubated with anti-KAISO (1:1000) overnight and with anti-goat immunoglobulin G and peroxidase for 30 minutes at room temperature. Slides were developed using 3,3´-diaminobenzidine/H2O2 (Dako, Denmark) and a hematoxylin counterstain. Slides were analyzed and photographed with a Nikon Eclipse E600 microscope.

### Statistical analysis

Data are expressed as means ± standard deviation (SD). The significance of differences between control and treated groups was evaluated using one-way analysis of variance (ANOVA). Experimental tests were performed at least three times. Differences were considered to be significant when P < 0.05.

## Results

**Figure 1:**
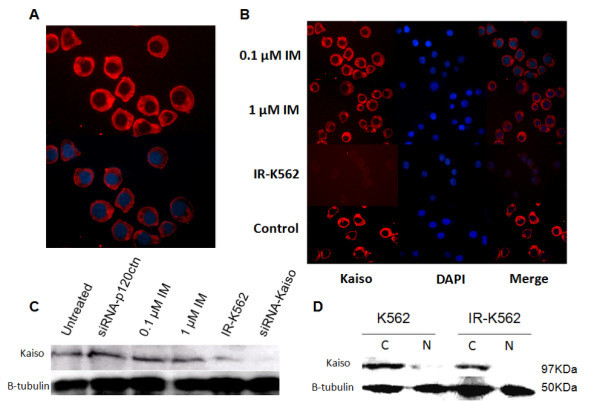
**Kaiso subcellular localization in K562 cell line (A). Immunofluorescence staining of Kaiso**. Kaiso was distributed in the cytoplasm, with less visible staining in the nucleus (**B**). Immunofluorescence of Kaiso in imatinib-resistant K562 cells and K562 cells treated with imatinib. (**C**). Expression analysis of Kaiso by immunoblotting assay. Normal bands can be detected after transfection with siRNA-p120ctn and treatment with imatinib. Little bands can be detected after transfection with siRNA-Kaiso and imatinib-resistant K562 cells. β-tubulin served as an internal control. (**D**). To verify effective separation of the cytoplasmic and nuclear fractions, cytoplasmic and nuclear extracts were immunoblotted for Kaiso in normal (K562) and imatinib-resistant K562 (IR-K562) cell line.

1. Kaiso: Cytoplasmic distribution of CML-BP.The studies in lung cancer have confirmed a cytoplasmic localization of Kaiso and associated with a poor prognosis of the patient [36,41]. To date, there is no evidence for the involvement of Kaiso in CML-BP. So we started by characterizing its subcellular distribution in K562 cell line since it has been considered as a cellular model of CML-BP. Being a more advanced phase of CML and has a poor prognosis for the patient, since some of them are resistant to imatinib therapy [3,7], it seemed appropriate to begin to characterize these cells.Immunofluorescence analysis showed the cytoplasmic distribution/accumulation of Kaiso in K562 cell line (Figure1A). A halo of expression can be clearly observed around the nucleus, involving the whole cytoplasm. For clarifying whether the subcellular distribution of Kaiso in K562 cells correlates with BCR/ABL activity, connecting Kaiso directly to CML, we performed inhibition of BCR/ABL by imatinib after 16 h of treatment. The immunofluorescence labeling of kaiso showed its presence predominantly in the cytoplasm of K562 cells administered with imatinib (Figure1B). In K562 cells treated with imatinib, β-tubulin was also mainly in the cytoplasm (data not shown). Kaiso labeling was not found in the K562 cells incubated with non-immune serum (data not shown).

To confirm the cytoplasmic localization of Kaiso in CML-BP, we analyzed cytoplasmic expression of Kaiso protein by western blot analysis, comparing expression in cytoplasmic and nuclear protein extracts in K562 cell line (Figure1D) and imatinib-resistant K562 cell line (Figure1D). Significant cytoplasmic expression of Kaiso was only observed in K562 cell line whereas in imatinib-resistant K562 cell line was clearly down-regulated (Figure1D). We also confirmed the weak expression of Kaiso in imatinib-resistant K562 cell line by immunofluorescence (Figure1B). Also by western blot, we confirmed that treatment with imatinib (0.1 and 1 uM) and siRNAp120ctn, did not disturb the expression of Kaiso (Figure1C).

**Figure 2:**
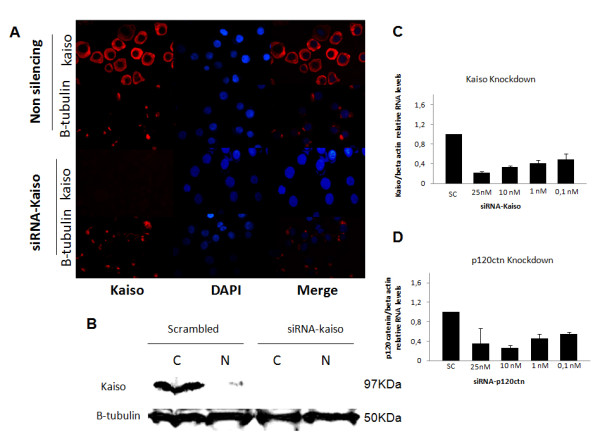
**siRNA-Kaiso efficiently down-regulates cytoplasmic Kaiso expression in K562 cell line**. (**A**). Immunofluorescence staining of Kaiso and β-tubulin. siRNA-Kaiso only efficiently downregulated cytoplasmic Kaiso expression in k562 cell line when compared to the scrambled knock-down cells (**B**). Cytoplasmic vs. nuclear fractionation Western blot analysis confirmed the results of immunofluorescence staining. β-tubulin served as an internal control. (**C**). Expression analysis of Kaiso knock-down by Real Time RT-PCR. (**D**). Expression analysis of p120ctn knock-down by Real Time RT-PCR. Data were expressed as mean ± standard deviation (S.D.). Columns, mean (n = 3); error bars, S.D.

2. RNAi knock-down of kaiso in K562 cells improves survival and proliferation.Given that Kaiso is overexpressed in the cytoplasm of K562 cells, this study set out to examine how loss of Kaiso and their partner p120ctn affected gene expression and cell proliferation of CML-BP. To inactivate Kaiso and p120ctn we employed siRNA targeting each gene as described in the materials and methods. We developed a transfection protocol that led to over 96% of the K562 cells taking up the siRNA (data not shown). Next, the effectiveness of the knockdown was assessed using QRT-PCR (Figure2C) and Western blotting (Figure2B). QRT-PCR analysis showed that Kaiso mRNA levels were decreased by 80% (Figure2C) and Western blot analysis showed that Kaiso protein levels were undetectable in K562 cells transfected by siRNA-Kaiso (25 nM for 24 h), when compared to scrambled knock-down cells (Figure2B). This result was confirmed by immunofluorescence in K562 cells transfected by siRNA-Kaiso, showing the undetectable expression of Kaiso (Figure2A). Using siRNA-p120ctn (10 nM for 24 h) a reduction of 70% in p120ctn was achieved when compared to scrambled knockdown cells by QRT-PCR analysis (Figure2D).

**Figure 3:**
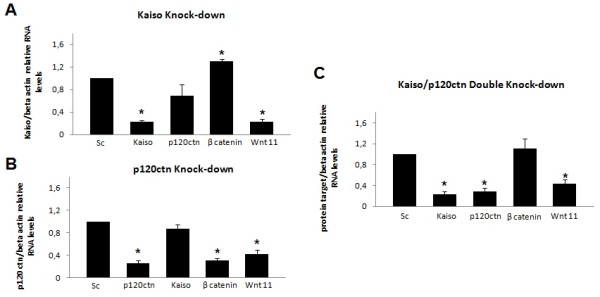
**Expression analysis of Wnt11, β-catenin, Kaiso and p120ctn genes in either Kaiso (A) or p120ctn (B) alone or in combination (C) knock-down cells**. K562 cells were transfected with the indicated siRNA combinations. Twenty-four hours later, RNA was isolated and subjected to Real Time RT-PCR to quantify expression of Wnt11, β-catenin, Kaiso and p120ctn after normalization to β-actin and compared to the scrambled knock-down cells. Data were expressed as mean ± standard deviation (S.D.). Columns, mean (n = 3); error bars, S.D.; *, p < 0.001.

To confirm these results, we analyzed the expression of two known Kaiso target genes, Wnt11 and β-catenin, using QRT-PCR. Wnt11 and canonical Wnt/β-catenin signaling pathway are modulated by Kaiso. K562 cells were either transfected with siRNA-scrambled that does not target any human gene or transfected with siRNA to Kaiso or p120ctn either alone or in combination. Knockdown of Kaiso led to significant increases by 13% in β-catenin gene expression (Figure3A). However, the p120ctn knock-down alone showed a decrease by 65% in β-catenin levels (Figure3B) while the Kaiso/p120ctn double knock-down line did not substantially affect β-catenin levels in vitro when compared to scrambled knock-down cells (Figure3C). Knock-down either Kaiso or p120ctn alone or in combination led to significant reduction of Wnt11 when compared to scrambled knock-down cells (Figure3). As is well known that Kaiso interacts with TCF/LEF1 [15,17], and that the Wnt11 promoter, has regulatory sites for binding TCF protein [42], these results suggest the inhibitory role of TCF/LEF1-β-catenin on the expression of Wnt11. In K562 cells transfected by siRNA-p120ctn, Kaiso may be responsible for Wnt11 repression (Figure3C).

**Figure 4:**
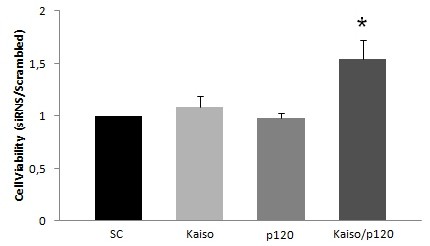
**Down-regulated cytoplasmic Kaiso and p120ctn enhance the proliferative ability of K562 cells**. Cell proliferation was determined using the WST-1 assay. The growth rate was significantly higher in siRNAKaiso/p120ctn double knock-down, when compared to scrambled knock-down cells. Data were expressed as mean ± standard deviation (S.D.). Columns, mean (n = 3); error bars, S.D.; *, p < 0.001.

**Figure 5:**
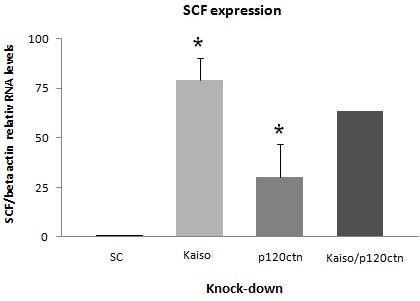
**Expression analysis of SCF gene by Real Time RT-PCR in either Kaiso or p120ctn alone or in combination knock-down cells**. Data were expressed as mean ± standard deviation (S.D.). Columns, mean (n = 3); error bars, S.D.; *, p < 0.001.

Since Kaiso is considered a methylation-dependent “opportunistic” oncogene, it was conceivable to explore the biological role of Kaiso on the cells growth in vitro, the proliferation of K562 cells was evaluated by a WST-1 assay. To knock-down either Kaiso or p120ctn alone or in combination, we employed siRNA. While the Kaiso knock-down alone did not show a substantial increase proliferation, the double knock-down showed a significant increase by 51% in proliferation, when compared to scrambled knock-down cells (Figure4). However, knock-down of p120ctn alone does not affect proliferation, when compared to scrambled knock-down cells (Figure4). Consistent with this finding, knock-down of either Kaiso or p120ctn alone or in combination, in K562 cells, led to a significant 10–100 fold increase in SCF expression assessed by QRT-PCR (Figure5). This significant increase in SCF expression correlated with an increase on in vitro cell proliferation.

**Figure 6:**
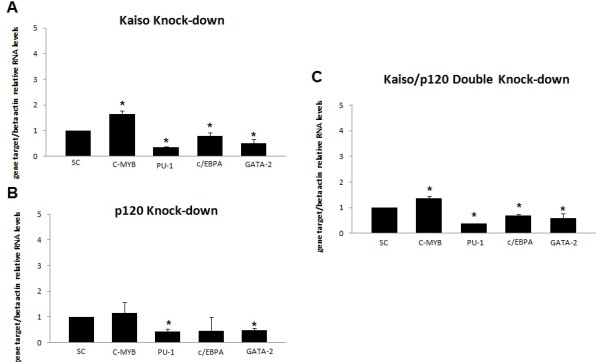
**Kaiso knock-down inhibits cell differentiation**. K562 cells were transfected with the indicated siRNA combinations. Twenty-four hours later, RNA was isolated and subjected to Real Time RT-PCR to quantify expression of C-MyB, PU-1, C/EBPalpha and Gata-2 after normalization to β-actin and compared to the scrambled knock-down cells. Data were expressed as mean ± standard deviation (S.D.). Columns, mean (n = 3); error bars, S.D.; *, p < 0.001.

3. RNAi knock-down of kaiso in K562 cells block hematopoietic differentiation.It was previously shown that Wnt11 can modulate hematopoietic stem cell diversification [43]. As mentioned above, knock-down of either Kaiso or p120ctn alone or in combination led to a significant reduction by 80% in Wnt11 expression. Our next step was investigate how loss of Kaiso and p120ctn, by siRNA, affected the cell differentiation status of CML-BP. We quantified the levels of hematopoietic differentiation genes: C/EBPα, c-Myb, GATA-2, PU.1, by QRT-PCR analysis. The knock-down of Kaiso alone or Kaiso/p120ctn double knock-down, increased c-MyB by 65% and decreased PU-1, C/EBPα and Gata-2 by 66%, 80% and 50% respectively, when compared to scrambled knock-down cells (Figure6A and C). The knock-down of p120ctn alone decreased PU1 and Gata-2 by 57% and 51% respectively when compared to scrambled knock-down cells (Figure6B). This leads us to think that the effect of knock-down Kaiso and p120ctn would block cell differentiation and increase proliferation of cells simultaneously in CML-BP.

**Figure 7:**
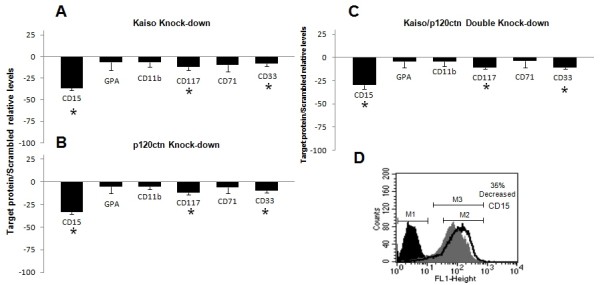
**Knocking-down expression of Kaiso in K562 cells reduced monocyte-macrophage differentiation**. Flow cytometric measurement of granulocytic specific surface antigen CD15 and CD11b in K562 cells after normalization with the scrambled knock-down expression. K562 cells were transfected with the indicated siRNA combinations **A**, **B** and **C**. The CD117 (c-Kit receptor), CD33 and the erythroid markers GPA and CD71 were also analyzed. (**D**) CD15 expression in K562 knockdown cells. M1 represents unlabeled K562 scrambled control. M2 represents K562 scrambled control labeled with CD15 and M3 represents K562 cells transfected with Kaiso duplex (10 nM) after 50 h-incubation. Data were expressed as mean ± standard deviation (S.D.). Columns, mean (n = 3); error bars, S.D.; *, p < 0.001.

We next investigated whether knock-down either Kaiso or p120ctn alone or in combination affects the global cell differentiation, now evaluating the maturation markers of hematopoietic differentiation CD15, CD11b, CD33 and CD117 expressed in the plasma membrane of K562 cells by FACS analysis. CD15 and CD11b were used widely as indicators of maturation of the hematopoietic cells and also as granulocytic markers [40]. We found that knock-down of Kaiso or p120 alone or Kaiso/p120ctn double knock-down decreased CD15, CD33 and CD117 by 25-35%, 8% and 13% respectively (Figure7). These finding indicate that knock-down of Kaiso and p120ctn are blocking the differentiation program of CML-BP. Finally, the down regulation of Kaiso and p120ctn decreased CD117 by 13% which is quite expected from the large amount of SCF expression (Figure5), suggesting down regulation of cell surface CD117/KIT receptors by an autocrine signaling mechanism.

**Figure 8:**
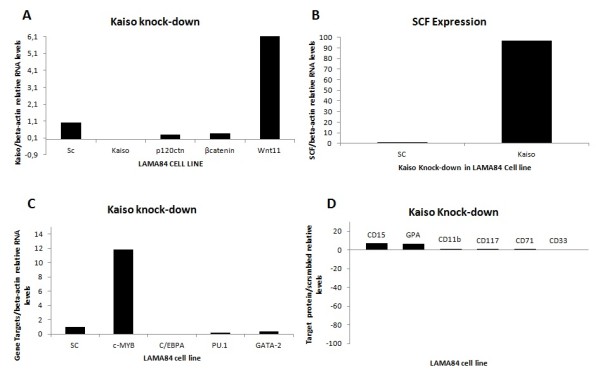
**Effect of RNAi knock-down of Kaiso on LAMA-84.** (**A**) Kaiso knock-down induce Wnt11 expression. LAMA-84 cells were transfected with 20nM of siRNA. Twenty-four hours later, RNA was isolated and subjected to Real Time RT-PCR to quantify expression of Wnt11, β-catenin, Kaiso and p120ctn after normalization to β-actin and compared to the scrambled knock-down cells. Columns, mean (n= 2). (**B**) Kaiso knock-down improve SCF expression in LAMA-84. Expression analysis of SCF gene by Real Time RT-PCR in Kaiso knock-down cells. Columns, mean (n= 2). (**C**) Effect of Kaiso knock-down on cell differentiation. LAMA-84 cells were transfected with 20nM of siRNA. Twenty-four hours later, RNA was isolated and subjected to Real Time RT-PCR to quantify expression of C-MyB, PU-1, C/EBPalpha and Gata-2 after normalization to β-actin and compared to the scrambled knock-down cells. Columns, mean (n= 2). (**D**) Knocking-down expression of Kaiso in LAMA-84 cells induced monocyte-macrophage differentiation. Flow cytometric measurement of granulocytic specific surface antigen CD15 and CD11b in LAMA-84 cells after normalization with the scrambled knock-down expression. LAMA cells were transfected with 20nM of siRNA after 50 hr-incubation. The CD117 (c-Kit receptor), CD33 and the erythroid markers GPA and CD71 were also analyzed. Columns, mean (n= 2).

In order to confirm the molecular analysis in K562 we used another CML-BP cell line, LAMA-84 (Figure8).The main difference between the cell lines K562 and LAMA-84 is the expression of β-catenin in response to the Kaiso knock-down. The knock-down of Kaiso increased β-catenin by 13% in K562 cell line (Figure 3A) and decreased by 62% in LAMA-84 cell line (Figure 8A) when compared to scrambled knock-down cells. This different behavior can be explained because LAMA-84 and K562 are cells in blast crisis, but with different origins. LAMA-84 is a human leucocytic cell line with basophilic characteristic [39] and K562 is a erythroblastic cell line with granulocytic and erythroid characteristics [38], besides being very much more differentiated than LAMA-84. 

**Figure 9:**
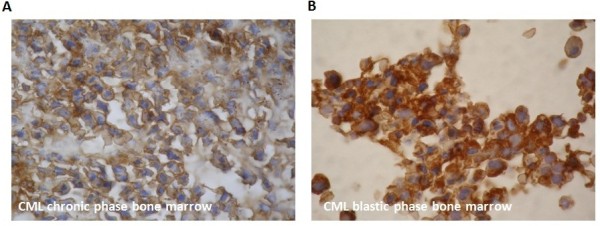
**Immunohistochemistry staining of Kaiso (A) and (B) in CML bone marrow slides, from blastic and chronic phase patients.** Magnification x 400 fold.

Finally to confirm the cytoplasmic localization of Kaiso, by immunohistochemistry, we compared their expression in CML bone marrow from patients in chronic and in blastic phase. Kaiso was expressed in the cytoplasm of the two compared phases (Figure9) and it can be argued that their cytoplasmic expression is significantly higher in blastic phase.

## Discussion

### Kaiso and cancer

The Kaiso protein, like other members of the subfamily POZ-ZF [20,22,25,44-48], has been implicated in cancer development process when it has been found that Kaiso inhibits activation mediated by β-catenin of the Mmp7 gene (also known as matrilysin), which is well known for metastatic spread [14]. Recently another study suggests that Kaiso can regulate TCF/LEF1-activity, via modulating HDAC1 and β-catenin-complex formation [15]. This shows that Kaiso can directly regulate the signaling pathway of canonical Wnt/β-catenin widely known for its involvement in human tumors. The Kaiso overexpression decreases the ability of TCF/LEF to interact with β-catenin, which implies that Kaiso and TCF/LEF are associated in the nucleus [17].

### Kaiso and prognosis

As expected for a transcriptional factor, the Kaiso protein is often found in the nucleus of several tumor or non-tumor derived mammalian cell lines [28]. Recent studies using immunohistochemistry analysis of normal and tumor tissue revealed that Kaiso protein is predominantly localized in the cytoplasm of the cell or is totally absent, though [49]. These data are consistent with the results found in the K562 cell line in which expression of the Kaiso is predominantly cytoplasmic (Figures 1 and 2). This seems to be unusual because Kaiso has a signal “NLS” highly conserved and required for any protein with nuclear localization. Moreover, Kaiso uses classical nuclear transport mechanisms through interaction with Importin α/β nuclear [50]. One possible explanation is that Kaiso, like other proteins or factors that normally reside in the cytoplasm, require a post-translational modification, to be targeted and translocated to the cell nucleus.

However, 2009 data has shown for the first time that the subcellular localization of Kaiso in the cytoplasm of a cell is directly associated with the poor prognosis of patients with lung cancer (non-small cell), and around 85 to 95% of lung cancers are non-small cell [36]. Such data shows a direct relationship between the clinical profile of patients with pathological expression of Kaiso.

Surprisingly in this paper we describe for the first time a relationship between the cytoplasmic Kaiso to CML-BP. An interesting aspect of our results is the relationship between cytoplasmic Kaiso to the prognosis expected in blast crisis (Figure2). At this stage of the disease, many patients died between three and six months, because they are refractory to most treatments. In CML progression to accelerated phase and blastic phase appears to be due mainly to genomic instability, which predisposes to the development of other molecular abnormalities. The mechanisms of disease progression and cytogenetic evolution to blast crisis remain unknown.

### Canonical and non-canonical Wnt pathways regulation of Wnt 11

The Wnt11 promoter contains two conserved TCF/LEF binding sites (at −43 for each of the alternative first exons) and one Kaiso binding site (at −775 in the human gene), suggesting that both canonical [42] and non canonical Wnt pathways can down regulate Wnt11 transcription directly. Consistent with this, Kaiso depletion strongly increase Wnt11 expression in Xenopus [29]. On the contrary, in K562 cells, upon Kaiso knock-down we observed a significant decrease in the Wnt11 expression (Figure3A–C). A possible explanation of this controversy is that knock-down of Kaiso, increased β-catenin expression (Figure3A), and this is a likely reason for the maintenance of Wnt11 repression in the absence of Kaiso. As is well known, Wnt11 is actually one of several β-catenin/TCF target genes that contain adjacent putative Kaiso and TCF/LEF binding sites in their promoter, suggesting that Kaiso and TCF/LEF cooperate to repress Wnt11transcription [16].

Our results therefore indicate the cooperation between β-catenin/TCF and Kaiso/p120ctn in negative regulation of Wnt11. A common theme among all these studies is that while Wnt11 expression can be regulated by canonical Wnt signals, this regulation is highly dependent on transcription factors in addition to, or other than, TCF/LEF family members, for example, Kaiso/p120ctn.

### Kaiso and resistance to imatinib therapy

The novel anticancer agent, imatinib (Glivec, Gleevec, formerly STI571, CGP57148) has proven to be a highly promising treatment for CML. The drug selectively inhibits the kinase activity of the BCR/ABL fusion protein [4,5].

Although the majority of CML patients treated with imatinib show significant hematologic and cytogenetic responses, resistance to imatinib is clearly a barrier to successful treatment of CML patients. In some patients, resistance arises due to powerful selective pressure on rare cells that carry amplified copies of the ***BCR-ABL*** fusion oncogene or point mutations in the BCR-ABL tyrosine kinase domain that affect binding of the drug to the oncoprotein. However, in a proportion of patients neither mechanism operates, and resistance appears to be a priori, existing prior to exposure to the drug [51]. These mechanisms of imatinib resistance are poorly understood and heterogeneous involving largely BCR-ABL independent mechanisms [51,52].

Our results show that imatinib-resistant K562 cells has a weak expression of Kaiso in the cytoplasm and with a similar phenotype, but not identical, to Kaiso knock-down cells (Figure1B–D). This result suggests the down regulation of Kaiso as a mechanism of resistance to imatinib. Obviously cannot rule out that weak expression in the imatinib-resistant K562 cell line, is a secondary effect involving other genes that lead to transcriptional and translational repression of Kaiso. So far, no proteomics studies, using high-throughput technologies, identified Kaiso as a gene potentially involved in the acquisition of resistance to imatinib [53,54].

### Extensive changes in gene expression underlie the biological effects of Kaiso knock-down

The result shows a global change affecting the expression of several genes important in hematopoietic differentiation and proliferation, coherently with the genome-wide transcriptional response to Kaiso, characterized during early vertebrate development [55]. Thus, all the changes produced by siRNA indicate a trend towards improvement of cell proliferation and blocks of granulocytic differentiation.

### Kaiso knock-down improves cell proliferation

The knock-down of either Kaiso or p120ctn alone or in combination decreased C/EBPα and PU-1 (Figure6A and C) and increased significantly SCF expression (Figure5). The transcription factor CCAAT/enhancer binding protein α (C/EBPα) is a strong inhibitor of cell proliferation [56]. Accordingly we found that in all transfections, C/EBPα levels were reduced by 56-80%, when compared with scrambled knock-down cells.

On the other hand, the transcription factor PU.1 is a hematopoietic lineage- specific ETS (Erythroblast Transformation Specific) family member [57] that is absolutely required for normal hematopoiesis [58]. The level of PU.1 expression is critical for specifying cell fate, and, if perturbed, even modest decreases in PU.1 can lead to leukemias and lymphomas [57,59-64]. Coherently, our results showed that the PU-1 levels decreased by 57-66% when either Kaiso or p120ctn alone or in combination levels were decreased by siRNA (Figure6).

An important aspect of our analysis is that recent data show a system of autocrine and paracrine activation of c-kit (CD117) by SCF [65]. These mechanisms stimulate the growth of Merkel cell carcinoma in vitro. Analysis of the expression of c-kit on the surface of K562 cells showed a small but significant reduction of the CD117 receptor expression in cells with knock-down of either Kaiso or p120ctn alone or in combination (Figure7). On the other hand, Kaiso/p120ctn double knock-down led to a significant 100 fold increase in SCF expression, important for cell survival and proliferation (Figure5). These results could represent an indirect evidence of autocrine and paracrine stimulation of c-kit in K562 cells and justify the effect on cell proliferation produced by Kaiso/p120ctn double knock-down.

### Kaiso knock-down inhibits cell differentiation

Recent studies demonstrate that Kaiso and N-CoR (interacts directly with Kaiso in the nucleus) have important roles in neural cell differentiation [35,66,67]. Also, the POZ-ZF subfamily member BCL6 represses several genes that are necessary for the terminal differentiation of B-lymphocytes [68]. But there is no evidence to support the participation of Kaiso in the hematopoietic differentiation.

Our results showed that knock-down of Kaiso decreased CD15 by 35%, indicating that, reduced expression of Kaiso, can block differentiation of the granulocytic program (Figure7D). We also analyzed the levels of Wnt11, C/EBPα and c-MyB and the results in Figure6 show that the expression of Wnt11 and C/EBPα were also reduced and the expression of c-MyB was increased, which is consistent with the Kaiso contribution to the hematopoietic differentiation.

A major role for Wnt11 in vivo is its ability to promote differentiation, for example, stimulating cardiac differentiation of mouse embryonic carcinoma P19 cells [69], and promoting differentiation of many different types of cells [70-72]. Moreover, Wnt11 promote the differentiation of QCE6 cells into red blood cells and monocytes at the expense of macrophages [43], suggesting that Wnt11 can modulate hematopoietic stem cell diversification. Thus, the knock-down of Kaiso decreased Wnt11 levels by 78% (Figure3), consistent with the role of Kaiso in the hematopoietic differentiation program.

On the other hand, knock-down of Kaiso reduced C/EBPα that is a critical regulator of hematopoietic stem cell homeostasis and myeloid differentiation [73,74]. The events leading to the loss of C/EBPα function facilitate leukemogenesis by blocking granulocytic differentiation [75,76] and coherently the knock-down of Kaiso decreased CD15 used widely as granulocytic marker (Figure7A–D).

Interestingly, in vitro experiments have shown that constitutive overexpression of c-Myb blocks differentiation of myeloid and erythroid cells and the associated growth arrest that occurs with maturation. However, c-myb-antisense-treated HL-60 cells differentiated only into monocytes but not into granulocytes indicating that granulocytic differentiation, unlike monocytic differentiation, requires c-myb-mediated proliferation. Consistent with this, an increase expression of c-MyB resulted in a significant decrease in expression of CD15 in K562 cells transfected with siRNA-Kaiso (Compare Figures 6A and 7A). Finally, the myeloid commitment of hematopoietic progenitors is characterized by the progressive loss of CD34 expression accompanied by the acquisition of CD33 expression at high levels. The knock-down of Kaiso led to a significant decreased by 8% in CD33 expression (Figure7).

These findings provide a comprehensive picture of the changes in proliferation, differentiation, and global gene expression that underlie of the pivotal role of cytoplasmic Kaiso in the blast crisis.

## Conclusions

Our results are promising first because they allow the establishment of relationship between blast crisis to cellular distribution of Kaiso, and second, by the extensive changes in gene expression underlie the biological effects of Kaiso knock-down and third because the epigenetic regulation of Kaiso make CML a particularly attractive disease for epigenetic drug targets.

Although the epigenome offers promising targets for novel anticancer therapy, an important obstacle still need to be considered. Where is Kaiso in the cytoplasm? What is the role of endocytic membrane in the disease progression? It is now widely accepted that systems of endocytic membrane trafficking and intracellular signaling are closely interconnected and endosomes could act as signaling platforms [77,78].

Therefore, a view focused on subcellular compartments and proteins modulating the epigenoma, can provide a greater understanding of the biology of malignant cells, as well as improve our approach to cancer treatment [78]. It is known that cancer treatment is dictated by the stage of the disease, and that cancer treatment is more effective during the chronic phase of the disease. Unfortunately, clinical and molecular tests cannot predict disease progression, which can create an obstacle to diagnosis: the inability to identify subtypes of patients most likely to benefit from specific treatment options for specific stages of the disease, which would make it possible to offer a therapy targeted to a given cancer patient. The results presented in this work reveal Kaiso and their subcelular distribution as a potential target for selective therapy of CML.

The understanding of this new biology of CML progression can provide markers for clinical diagnosis and different approximations for better therapeutic strategies.

## Competing interests

The authors declare that they have no competing interests.

## Authors’ contributions

JC conceived of the study, carried out the Kaiso/p120ctn knockdown studies and performed the molecular analysis in K562, and drafted the manuscript. JM performed the immunofluorescence analysis. LP carried out the Kaiso/p120ctn knockdown studies and performed the molecular analysis in LAMA-84, and carried out the immunohistochemical analysis in patients. EA conceived of the study, participated in its design and coordination. All authors have read and approved the final manuscript.
